# Food Security and Land Use under Sustainable Development Goals: Insights from Food Supply to Demand Side and Limited Arable Land in China

**DOI:** 10.3390/foods12224168

**Published:** 2023-11-18

**Authors:** Yang Lan, Bingjie Xu, Yizhong Huan, Jinhua Guo, Xiaojie Liu, Jingwen Han, Keran Li

**Affiliations:** 1 Key Laboratory of Natural Resource Coupling Process and Effects, Ministry of Natural Resources, Institute of Geographic Sciences and Natural Resources Research, Chinese Academy of Sciences, Beijing 100101, China; ucbq737@ucl.ac.uk (Y.L.); guojinhua871001@126.com (J.G.); 2 College of Land Science and Technology, China Agricultural University, Beijing 100193, China; xubingjie21@mails.ucas.ac.cn (B.X.); hanjw@cau.edu.cn (J.H.); 3 The Bartlett School of Environment, Energy and Resources, University College London, London WC1E 6BT, UK; 4 Resources Geography and Land Resources Research Division, Institute of Geographic Sciences and Natural Resources Research, Chinese Academy of Sciences, Beijing 100101, China; 5 College of Resources and Environment, University of Chinese Academy of Sciences, Beijing 100049, China; 6 College of Humanities and Development Studies, China Agricultural University, Beijing 100083, China; yizhonghuan@gmail.com; 7 College of International Development and Global Agriculture, China Agricultural University, Beijing 100083, China; 8 College of Energy, Chengdu University of Technology, Chengdu 610059, China; keranli98@outlook.com

**Keywords:** food security, food production, food consumption, arable land, land demand

## Abstract

The conflict between economic growth and the arable land demand poses a significant challenge to maintaining food security and achieving the Sustainable Development Goals. Meanwhile, substantial regional disparities in food consumption contribute to variations in land demand, further exacerbating constraints on food security. However, few studies have delved into regional differences in land demand related to food consumption. To bridge these gaps, this study estimated the arable land demand and associated pressures, considering food consumption patterns and the land footprint across 31 provincial districts in China. The findings reveal that grains remain the primary crop consumed by Chinese residents. Notably, the food consumption pattern exhibits substantial disparities among provincial districts, particularly concerning livestock products. Given China's vast population and escalating consumption of livestock, the country demonstrates heightened land demands. While China does not face a national-level food security threat, regional disparities are evident, with eight provincial districts facing potential food security risks. This study explored the challenges and pathways in maintaining food security and the visions to achieve it, emphasizing the importance of sustaining a balanced food consumption pattern, reducing food waste, improving environmentally friendly agriculture practices, formulating effective and continuous laws and regulations, and exploring potential land resource development to alleviate the pressure on arable land and ensure food security.

## 1. Introduction

Human survival and development are global issues. To cope with the environmental crisis and the imperative for sustainable development, the Sustainable Development Goals (SDGs), long-term goals aimed at guaranteeing the right to basic survival and development for all people at all times, were first proposed in 2015 [1]. A key element in achieving the SDGs is food security and nutrition, serving as the cornerstone for attaining the broader goals outlined within the SDGs [2,3].

Food provides energy and nutrition to humans and is vital for human metabolism. Ensuring food security involves not only maintaining a sufficient and accessible food supply but also addressing the increasing nutritional needs of a growing population [4,5]. While global food production has increased by about 30% over the past four decades, the nearly doubled global population has outpaced this growth, resulting in a shortfall in current food production to meet the surging demand for food [6]. Meanwhile, van Dijk et al. have predicted that the total global food demand will increase by 35% to 56% from 2010 to 2050 [7]. This foreseen outcome poses a heightened risk to countries with limited food production capacity, making them more vulnerable in terms of food security due to the surging demand for food. In addition, food consumption varies greatly among nations, especially meat consumption. To be specific, developed countries (e.g., the United States and Australia) consume more than twice as much meat as developing countries (e.g., China and Mexico) [8]. These supply–demand gaps have led to a disparity in human nutrition levels worldwide. While more than one in eight adults is obese, particularly in developed countries [9], over 800 million people worldwide still suffer from malnutrition, and nearly 2.4 billion people face insufficient food access. The impact of COVID-19 has further exacerbated this issue, leading to an expanding number of malnourished individuals, despite a 50% decrease over the past two decades [10,11]. This global disparity in nutrition levels poses challenges to the realization of Goals 2 and 12 within the SDGs, both of which aim to guarantee food security [12].

Moreover, food security has profound implications for resource consumption and environmental protection [13]. Food consumption, especially livestock products, could increase due to population growth and the consumption pattern upgrade, posing a challenge to food security. For example, the water footprint per calorie for beef is, on average, 20 times larger than that for cereals and starchy roots [14]; the carbon footprint of livestock products significantly exceeds that of crops [15,16]; and the amount of arable land demand to provide the same amount of energy and protein to humans through meat products is four times that for vegetarian products [17]. Alexander et al. (2016) discussed the impact of the consumption pattern upgrade on global agricultural land, taking the dietary structures in the United States and India as examples. The results indicate that if the world were to adopt the US dietary structure, the arable land demand to meet the food demand would be approximately six times that of India [18]. Additionally, agrochemical-based agricultural production contributes to non-point-pollution sources, including runoff from cropland, wastewater (including urine and flushing water) and manure from livestock operations, and aquaculture wastewater (including residual feed and fish excrement) [19], causing serious environmental problems. Therefore, it is necessary to identify the relationship between food consumption, associated resource demands, and the environmental costs involved.

Arable land (AL), a limited and crucial resource for guaranteeing food security, serves as the foundation for food production. It is important to discuss the AL demand for food consumption and food security, sustainable land use, and environmental protection. The escalation in food consumption corresponds to an augmented demand for food consumption [20]. Meanwhile, the rapid urbanization process leads to a reduction in AL. For instance, among countries with more than 100 million ha of AL, the United States has an enormous AL reduction rate of 10.1%, followed by Russia and India at 2.8% and 2.2%, respectively [21]. To offset the reduction in grain production caused by the AL diminution, extensive crop rotation and high external inputs (e.g., biogeochemical flows) are applied to meet the demands for food. However, these measures have already extended sustainable planetary boundaries [22], giving rise to serious issues such as deforestation, soil pollution, and soil alkalization. To quantify the amount of AL demand for food production, Gerbens-Leenes et al. introduced the concept of virtual land and utilized it to calculate the land demand for food [23].

China, as the largest developing country, sustains 21% of the global population using only 9% of the world’s AL, grappling with substantial land pressures for food consumption. Meanwhile, China is undergoing profound transitions in its food consumption pattern, population growth, and urbanization process [24]. Notably, between 1980 and 2010, crop consumption in China decreased substantially, while livestock product consumption witnessed a noteworthy increase [25]. Scholars have calculated that from 1961 to 2013, China’s overall land demand for food consumption surged from 105 million ha to 175 million ha; the rise persisted as meat consumption increased, and the nation’s import dependence to meet food demand was also on the rise [26]. The urbanization process has brought about great changes in land use and occupied arable land for food production, intensifying land pressure [27]. Liu and Zhou mentioned that the spatial mismatch between water and soil resources and food production, along with the imbalanced spatial coupling of the population, land, and the food system, adversely impacts China's food security [28]. Meanwhile, the urbanization process has influenced residents' consumption habits within the fast-paced socio-economic transformation, indirectly altering residents' food consumption patterns [29]. Several studies have used the ecological footprint (EF) to estimate the land demand [30,31], in which Li pointed out that with the expansion of urbanization, the proportion of residents consuming food away from home increased, leading to a nearly 33-fold increase (44,440 ha) in the total EF in this sector from 2002 to 2015. Additionally, the vast territory and significant regional cultural differences in China contribute to variations in natural resource endowment, economic levels, and customs, resulting in differences in dietary habits, cultural backgrounds, and nutritional emphases among Chinese residents [32,33,34]. Thus, the food consumption patterns of Chinese residents exhibit substantial diversity among provinces, with their dietary structures often displaying imbalances.

To sum up, existing research on the transition of food consumption patterns in China has mostly focused on the virtual water and water footprints at the national scale [35,36]. Few scholars have delved into this subject from the perspective of land use at the inter-provincial scale. Therefore, a comprehensive examination of the land demand for food consumption, land footprint, and associated pressures in China at the provincial level is essential to bridge this research gap and guarantee China's food security. In response to this need, this study aims to address this knowledge gap by focusing on the spatial disparities in food consumption, land demand, and associated pressures at the provincial level in China using official statistics. Our approach involves three key steps. Firstly, we estimated China’s food consumption across 31 provincial districts in China from 2015 to 2018. Secondly, we calculated the land footprint of food, as well as the per capita and total AL demand for food consumption in each district, and analyzed land pressures for food consumption in different regions. Finally, herein, we summarize the challenges stemming from the transitions in food consumption patterns, population, productivity, and urbanization and provide recommendations to achieve food security. The outcomes of our study aim to elucidate the challenges of China’s food security due to food consumption and limited AL and contribute to predicting the land pressure on China's food security in 2050 so as to inform decision making for policy makers and stakeholders.

## 2. Materials and Methods

### 2.1. Study Area

**Figure 1:**
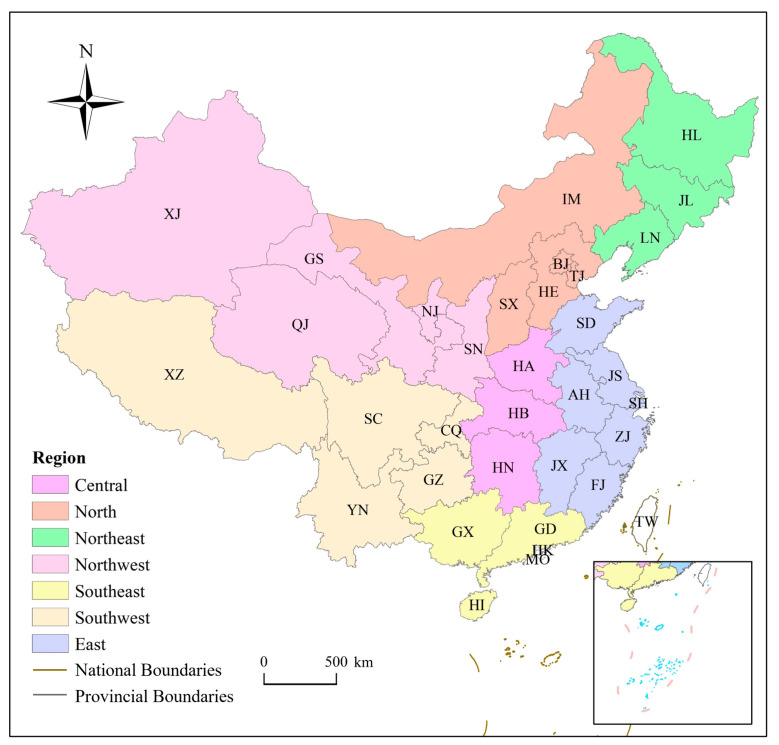
China’s provincial districts (including provinces, municipalities, and autonomous regions). Table A1 shows the corresponding full name of each provincial district. Source: Authors’ original creation.

This study focused on the Chinese mainland, excluding Hong Kong and Macao. This article divides the Chinese mainland into seven regions by geographical conditions and dietary habits (Figure 1).

### 2.2. Data Processing and Analytical Procedures

This study quantified land demand by virtual land use (VLU) and the ecological footprint (EF). VLU is defined as the land area used in the production of a unit of a food commodity [23], designed to calculate the land demand for food production. Calculating VLU requires the consideration of both food consumption and land productivity. On the other hand, the EF refers to the quantity of natural resources required to sustain people or the economy [37] and aims to illustrate the human impact on the environment, i.e., to calculate the environmental carrying capacity of human activities. The EF can be compared at the individual, regional, national, or global level and varies annually with the population, per capita consumption, and productivity. Currently, the EF has become one of the most widely used methods worldwide for benchmarking environmental performance and monitoring progress toward a sustainable future.

By employing these two concepts, land demand can be measured on both temporal and spatial scales and can be used in conjunction with demographics and other economic indicators to showcase food security within specific regions [38,39].

#### 2.2.1. Food Consumption

Food consumption (FC) is categorized into two types: crops, including grains (cereals, potatoes, and beans), vegetables, and fruits, and livestock products, including meats (pork, beef, mutton, and poultry), eggs, dairy products, and aquatic products.

This study collected data on food consumption in the urban and rural regions of each provincial district to estimate the mean FC for an entire provincial district. Food consumption for an entire provincial district can be calculated using Equation (1), as shown below:

(1) $${\mathrm{FCP}}_{i}=\frac{\left({\mathrm{FCUP}}_{i}\text{×}P_{U}+{\mathrm{FCRP}}_{i}\text{×}P_{R}\right)}{P_{T}}$$

FCP_i_, FCUP_i_, and FCRP_i_ refer to per capita FC of type i for one entire provincial district. P_U_, P_R_, and P_T_ are the urban permanent population, rural permanent population, and provincial total permanent population, respectively (T: total; U: urban; R: rural).

The Food and Agriculture Organization (FAO) provides detailed content information regarding calories, proteins, and fat for each food item. In this study, the caloric, protein, and fat intake of each provincial district were estimated by multiplying the caloric, protein, or fat contents of various foods by the FC value in each provincial district, as detailed in Equations (2)–(4).

(2) $${\mathrm{CalT}}_{i}={\mathrm{FCTP}}_{i}\text{×}{\mathrm{Cal}}_{i}$$

(3) $${\mathrm{ProtT}}_{i}={\mathrm{FCTP}}_{i}\text{×}{\mathrm{Prot}}_{i}$$

(4) $${\mathrm{FatT}}_{i}={\mathrm{FCTP}}_{i}\text{×}{\mathrm{Fat}}_{i}$$

CalT_i_, ProtT_i_, and FatT_i_ refer to the per capita intake of calories, protein, and fat from food (type i) in each provincial district. Cal_i_, Prot_i_, and Fat_i_ refer to the caloric, protein, and fat contents of food.

To assess the reasonability of the FC patterns in each province, this article refers to the balanced diet in the China Dietary Guidelines proposed by the Chinese Nutrition Society [40] (Table 1).

#### 2.2.2. Land Footprint of Food

Building upon the concepts of VLU and EF, this article introduces the term land footprint of food (LF), which is defined to estimate how much land is required to produce a unit of food, providing insights into agricultural productivity. It is important to note that higher agricultural productivity results in a lower LF, and this metric serves as an illustrative measure of the efficiency of agricultural land utilization.

As crops are produced directly from the land, the LF of crops can be directly estimated by Equation (5) as follows:

(5) $${\mathrm{LF}}_{i}=\frac{1}{Y_{i}}=\frac{s_{i}}{P_{i}}$$

Y_i_ refers to the unit yield of food (type i); P_i_ and s_i_ refer to the produced quantity of food (type i) and the sown area of food (type i), respectively; and LF_i_ refers to the land footprint of food (type i).

In addition, the production of livestock products depends on feed grains, so the land demand for livestock products can be estimated indirectly by introducing the feed-to-meat ratio (Table 2), a parameter representing the amount of grain consumed in producing 1 kg of livestock products. Therefore, the land footprint of livestock products can be estimated by Equation (6) as follows:

(6) $${\mathrm{LF}}_{i}=\frac{1}{Y_{g}}\text{×}t_{i}$$

Y_g_ and t_i_ refer to the unit yield of grain and the feed-to-meat ratio for livestock products (type i) in each provincial district, respectively.

#### 2.2.3. Land Demand for FC and Land Pressure of FC

Based on FC, the LF, and the population, this study estimated the land demand (LD) for FC using Equation (7), as detailed below:

(7) $${{\mathrm{LD}}_{T}=\mathrm{LD}}_{p}\text{×}P=\sum {\mathrm{FCTP}}_{i}\text{×}{\mathrm{LF}}_{i}\text{×}P$$

LD_P_ and LD_T_ refer to the per capita LD for FC and the total LD for FC, respectively. P refers to the permanent population.

Land pressure (LP) is determined by the land demand and existing land area, which can be characterized by the land pressure indicator of FC (LPI). The LPI is calculated as the ratio between the LD for FC and the sown area. Each provincial district has diverse AL types, including grain cropland, vegetable cropland, and orchard. It should be noted that the land demand for livestock products was estimated using grains, which should be included within the category of grain croplands.

This study estimated three types of ALs’ LPIs and the total LPI in each provincial district (Equation (8)). If LPI_T_ is <1, the AL in a district can meet the local FC demand; otherwise, this district should import food from other regions.

(8) $${\mathrm{LPI}}_{T}=\frac{{\mathrm{LD}}_{T}}{N_{T}}=\frac{\sum \mathrm{LP}I_{i}\text{×}N_{i}}{N_{T}}$$

where i and T refer to the different AL types and the total AL types, respectively. LD refers to the land demand for FC. N refers to the sown area for crops. The LPI refers to the land pressure indicator for FC.

### 2.3. Data Reliability

The dataset includes FC, land use, yield, and population. The values of per capita FC, yield, sown area, and permanent population were derived from the average values reported in the China Statistical Yearbook 2019 and the China Rural Statistical Yearbook 2019 for the period spanning 2015–2018 [44,45]. Parameters related to caloric, protein, and fat intake were based on the average values sourced from the FAO database over the same period, from 2015–2018 [41]. Recommended diet values were from the Chinese Dietary Guide [40]. The parameters of the feed-to-meat ratio are the average values obtained from the studies conducted by Xin and Tang and Li [42,43]. The average values from 2015 to 2018 were used as the dataset to enhance data accuracy and minimize bias.

## 3. Results

### 3.1. Food Consumption in China

**Figure 2:**
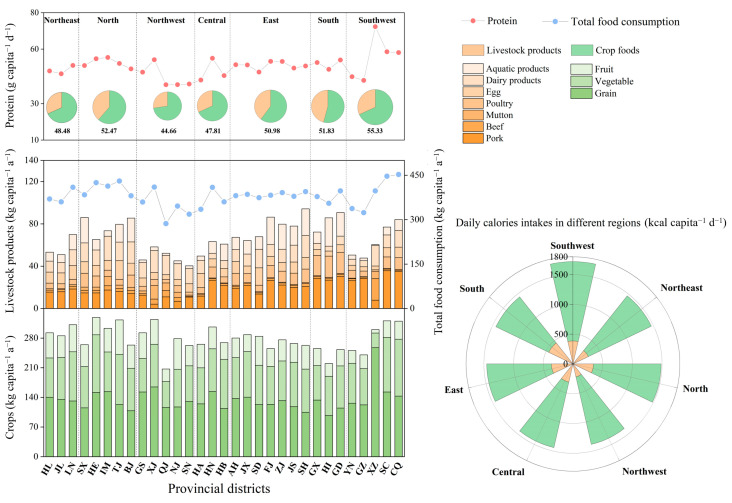
Per capita food consumption and caloric and protein intake in China, 2015–2018. Source: Authors’ original creation, data from China Statistical Yearbook 2019, and authors’ calculations.

From 2015 to 2018, China’s average FCP was 337.67 kg a^−1^, with corresponding average daily intakes of 1422.20 kcal of calories and 48.35 g of protein (refer to Figure 2). Notably, residents in developed regions, such as the south and the east, exhibited a tendency to consume more livestock products, vegetables, and fruits, while their grain consumption was comparatively lower.

Crops dominated the food consumption categories in all provincial districts, despite significant disparities in cultural practices, natural resource endowments, and socio-economic levels across different provincial districts. Grains, recognized for their higher caloric content compared to other food types, contributed 67% of the total caloric intake in China’s FCP. In relatively developed regions like South China, grain consumption was only 92% of the national average. The consumption of vegetables and fruits exhibited wide variations across provincial districts, reflecting local disparities in natural resource endowments. Per capita vegetable consumption ranged from 34 to 136 kg, while fruit consumption varied between 8 and 81 kg. Vegetable consumption in Tibet was only approximately 25% of that in other provincial districts, and per capita fruit consumption was merely 8.26 kg. Apart from being related to resource endowments, the consumption of fruit also demonstrated a certain correlation with the economic levels of different regions, with elevated levels observed in districts like Tianjin and Shandong.

Livestock product consumption constituted 20% of the total FC but was the primary source of protein (63%) and fat (77%), showing robust regional characteristics. Livestock product consumption varied in different regions, with notably higher levels observed in the relatively developed North, South, and East China. The livestock product consumption in East and South China was approximately 1.2 times higher than the national average. Pork was the predominant livestock product consumed in most provincial districts, contributing the most to protein and fat at 22% and 51% of the total intake, respectively. However, due to natural resource endowments and dietary habits, the per capita consumption of beef in Tibet and mutton in Xinjiang was higher, reaching 20.37 kg and 12.87 kg, respectively.

Regarding poultry and egg consumption, residents in Guangdong and Shandong consumed approximately 1.2 times and 1.5 times the national average, respectively. This disparity can be attributed to the substantial local production of poultry and eggs in these districts. Moreover, for aquatic and dairy product consumption, which is closely related to regional characteristics, people tended to consume more aquatic products in coastal regions like Fujian and Hainan and more dairy products in Xinjiang and Inner Mongolia, two northwest provincial districts.

### 3.2. Land Footprint in China

**Figure 3:**
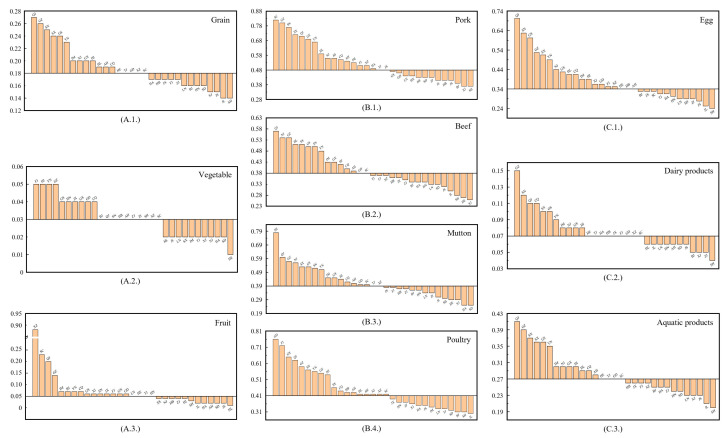
Land footprints of food in China (ha t^−1^). (**A.1.**) LF of grain, (**A.2.**) LF of vegetables, (**A.3.**) LF of fruit, (**B.1.**) LF of pork, (**B.2.**) LF of beef, (**B.3.**) LF of mutton, (**B.4.**) LF of poultry, (**C.1.**) LF of eggs, (**C.2.**) LF of dairy products, (**C.3.**) LF of aquatic products. Note: The intersection of the horizontal and vertical coordinates represents the national average land footprint for each type of food. Source: Authors’ original creation, data from China Rural Statistical Yearbook 2019, and authors’ calculations.

The LFs of livestock products were much higher than that of crops, indicating that producing a unit of livestock products requires more land (refer to Figure 3). Pork had the largest LF at 0.48 ha t^−1^, which was 17 times higher than that of vegetables (0.03 ha t^−1^). Poultry, beef, and mutton also exhibited high LF values. The LF was impacted by natural endowments and regional productivity levels, with eastern China having the lowest value due to better production conditions. The LF of grains was highest in the northwest, whereas it was the lowest in Jilin, a province in Northeast China, at 0.14 ha t^−1^. Apart from some provincial districts in the southwest, the LFs of vegetables and fruits in most districts were similar and indistinguishable based on production technology improvements. The LFs of livestock products, mainly impacted by large-scale farming, were lower in the central and the eastern regions, with the lowest value observed in Shanghai, falling below 76% of the national average.

### 3.3. Land Demand for FC in China

**Figure 4:**
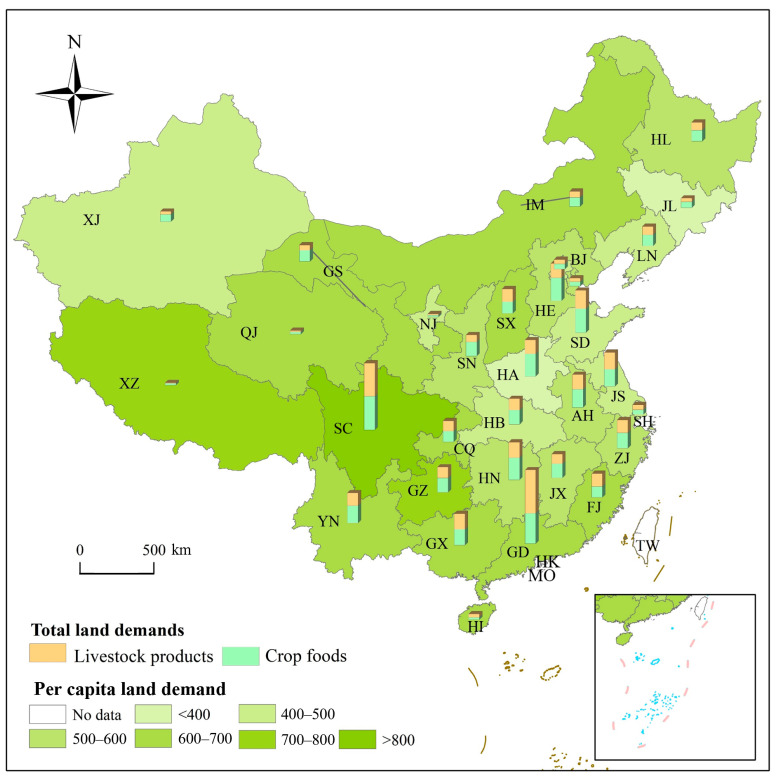
Spatial distribution of total land demand and per capita land demand in China. Source: Authors’ original creation, data from authors’ calculations.

Heterogeneity in productivity (LF), FC, and the population affect LD. Figure 4 shows the spatial differences in LD_T_ and LD_P_ in China. On the one hand, LF and FC play pivotal roles in shaping LD_P_. From 2015 to 2018, China’s average LD_P_ was 500 m^2^·a^−1^, of which 55% was for crop foods and 45% was for livestock products. Grains contributed to 45% of the total LD_P_, followed by pork and poultry. Despite the high consumption levels, LD_P_ for vegetables and fruits was only 27.46 m^2^·a^−1^ and 21.79 m^2^·a^−1^, respectively. LD_P_ surged in high-livestock-product-consumption regions (e.g., the south and the southwest), especially in districts with rapid urbanization processes and low agricultural productivity (e.g., Sichuan). Notably, LD_P_ in the east was not tremendous, while livestock product consumption was high, probably attributed to large-scale breeding practices that reduced LF. In contrast, despite a lower consumption of livestock products in the northwest, the harsh feeding conditions elevated LF and resulted in increased LD_P_, exemplified by Gansu, which had 1.3 times China’s average LD_P_. On the other hand, the population is a crucial factor in LD_T_. From 2015 to 2018, China’s LD_T_ amounted to 6.86 × 10^7^ ha·a^−1^, with 3.78 × 10^7^ ha·a^−1^ and 3.08 × 10^7^ ha·a^−1^ for crops and livestock products, respectively. Among livestock products, LD_T_ for beef and mutton accounted for only 2% of the total LD_T_, while LD_T_ for pork accounted for 20% of the total LD_T_, approaching the combined LD_T_ for other livestock products.

East and South China, characterized by more developed economic levels, larger populations, and higher consumption of livestock products, exhibited higher total land demands for food consumption, accounting for 45% of that in China. Among them, Guangdong, the province with the largest population, contributed nearly 11% of the total LD_T_ in China, whereas the sown area for croplands accounted for only 3.2%, signifying that Guangdong was facing a substantial food supply gap. The disparity in LD_T_ among the provincial districts in Southwest China was particularly pronounced. Sichuan, the most populous provincial district in the southwest, had the highest pork consumption in China. However, challenging terrain conditions reduced agricultural productivity, leading to exceptionally high land demand, reaching 6.77 × 10^6^ ha·a^−1^. In contrast, Tibet, with the smallest population, consumed very few livestock products, and crops accounted for the majority of the diet, resulting in LD_T_ accounting for only 3.5% of the national total.

### 3.4. Land Pressure for FC in China

**Figure 5:**
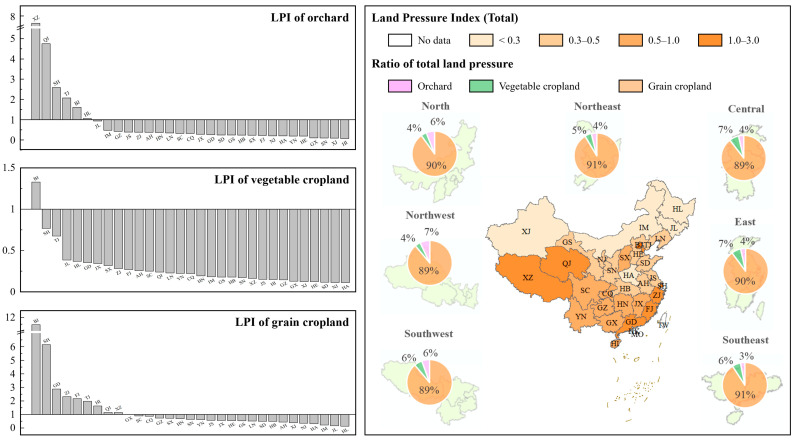
The index, contribution, and proportion of land pressure indicators in China. Source: Authors’ original creation, data from authors’ calculations.

Figure 5 illustrates the spatial values and differences in the LPI in China. From 2015 to 2018, China’s LPI_T_ was 0.46, indicating that food security was not a serious issue at the national level but was uneven at the regional level. For instance, LPI_T_ in Southwest and South China were considerably higher than those in other regions, which is related to the different agricultural conditions, resources, and populations across China. On the contrary, LPI_T_ was lower in Northwest and North China. In general, grain cropland contributed to about 90% of LPI_T_, whereas vegetable cropland and orchard contributed to 3–7% of LPI_T_, though the contribution of the three types of croplands in different regions had slight differences.

At the provincial level, eight provincial districts had LPI_T_ >1.0, indicating that FC in these districts could not meet the demand, posing potential food security risks. Most of these districts are in eastern China, known for higher levels of urbanization and economic development, where arable land is heavily occupied for urban construction. Consequently, these districts could consider importing food from other districts with low LPI_T_. Specifically, the LPI of grain cropland, a key indicator of food security, was >1.0 in nine provincial districts. Beijing had the maximum value (11.47) due to a limited sown area and a large population. Similar conditions were observed in Tianjin, Shanghai, and Guangdong. Tibet and Qinghai, in western China, also faced higher pressure on grain cropland (1.23 and 1.16, respectively) compared to other regions. Although Tibet and Qinghai possess abundant grain cropland resources, the inclement climate has slashed their productivity, contributing to an increase in grain cropland pressure. Nevertheless, the remaining provincial districts in China had a much lower grain cropland pressure. As for the other two croplands, only Beijing’s LPI of vegetable cropland was slightly >1, and six provincial districts had an LPI of orchard >1, particularly Tibet and Qinghai (7.64 and 4.76, respectively), which were mainly due to poor natural conditions and inefficient productivity.

### 3.5. Factors Affecting LP

#### 3.5.1. FC Pattern Transition

**Figure 6:**
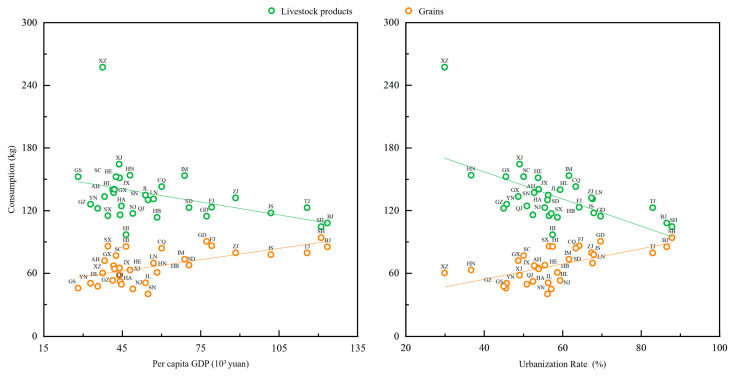
The relationship between per capita GDP and food consumption and the relationship between urbanization rate and food consumption in China, 2015–2018. Source: Authors’ original creation, data from China Statistical Yearbook 2019 [38] and authors’ calculations.

The level of the local economy and urbanization are among the key determinants of food consumption. With economic development and an increasing population concentration in cities, people tend to consume more meat and high-protein foods and less grain [46,47,48], gradually lowering the consumption ratio of grain to livestock products. This observation aligns with the results of this study (Figure 6 and Table 3). From 2015 to 2018, the consumption of livestock products in eastern and southern China was remarkably higher than that in Northeast China, where grain consumption was relatively high. Figure 6 elucidates the relationship between per capita GDP and food consumption, as well as the relationship between the urbanization rate and food consumption in China from 2015 to 2018. As per capita GDP and the urbanization rate increase, there is a diametrically opposite trend in the consumption of livestock products and grains: the former shows a positive correlation with per capita GDP or the urbanization rate, whereas the latter exhibits a negative correlation. This implies that the level of economic development and urbanization have a significant impact on the transition of food consumption patterns.

#### 3.5.2. Arable Land Productivity

**Figure 7:**
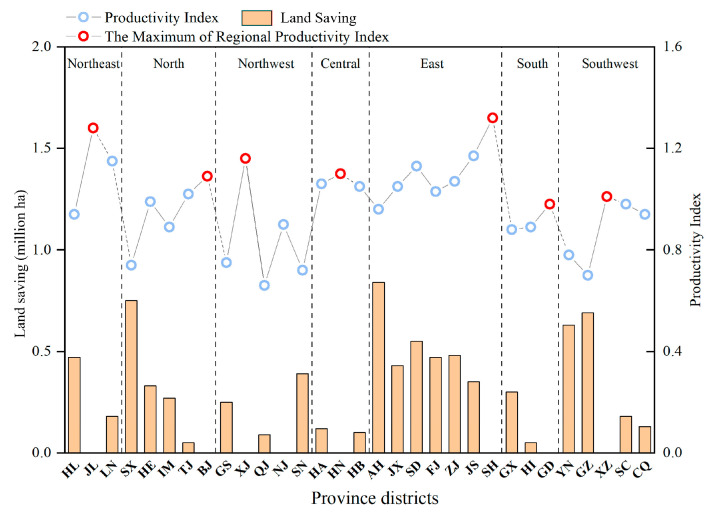
The productivity indices in provincial districts and the land saving when all of the provincial districts’ productivity is raised to the highest regional value. Source: Authors’ original creation, data from authors’ calculations.

Given the diversity of natural conditions and economic levels across different regions, we calculated the average grain cropland productivity index of all provincial districts in China from 2015 to 2018 and made the following assumption: the highest productivity in each of the seven regions in China represented the maximum standard achievable for all provincial districts within their respective regions. Figure 7 demonstrates the provincial districts with the highest grain cropland productivity index in each region, which were Jilin, Beijing, Xinjiang, Hunan, Shanghai, Guangdong, and Tibet (since the natural conditions in Xinjiang and Tibet were quite different from other provincial districts in the northwest and northeast, the productivity indices in Ningxia and Sichuan were taken as the standard values in their respective regions). If all of the provincial districts in China can elevate their productivity and meet the above standards, China’s total land demand will decrease by approximately 8.1 million ha, which would be equivalent to 5% of the national grain planting area. The additional grain productivity resulting from this improvement could fulfill the food consumption needs of an additional 162 million people, which is equal to sevenfold the population of Beijing or sixfold the population of Shanghai. Furthermore, this improvement in agricultural productivity would help alleviate land pressure in each provincial district. It is worth mentioning that Shanxi would achieve the most savings in land demands, followed by Guizhou and Anhui.

## 4. Discussion

### 4.1. Challenges to China’s Food Security from LD

FC pattern transition, productivity diversity, and population dynamics are the main factors affecting LD, posing challenges to China’s food security. Since China’s population is expected to remain at approximately 1.3–1.4 billion from 2020 to 2050 [49,50,51], this article does not discuss the demographic factors but only the first two factors. In our opinion, China is anticipated to confront a dual challenge in the future— experiencing both a surging demand in food consumption and a reduction in cropland supply.

On the demand side, the increase in residents’ purchasing power due to economic development and urbanization tends to drive people to consume more meat, more high-protein foods, and fewer grains [46,47,48]. This transition impacts FC patterns and LD, posing a severe threat to China's food security. From 1961 to 2013, China’s LD for FC increased by 1.7 times [26]. Predictions by Sheng and Song suggest that China's food consumption could be 1.33 times that of the 2015–2018 period by 2050 due to residents’ demand for high-value commodities such as meat and dairy products [52], and estimates from the OECD and FAO indicate that China is expected to account for 45% of the global pork consumption in the next decade and also dominate per capita consumption of beef, poultry, and mutton [53]. According to the above assumptions, if food loss and waste in the whole supply chain from production to consumption is considered at 27% of annual food production [54,55,56], China’s LPI will reach 0.78 in 2050, which implies that China's food security issues will face greater challenges in the future. Addressing issues related to food loss and waste, as well as finding sustainable approaches to meet changing consumption patterns, will be crucial for securing China's food future.

On the supply side, low-quality AL poses a challenge to the stability of the food supply. According to the 2019 National Cultivated Land Quality Grade Bulletin [57], only 31.24% of the total AL area in China is identified as high-quality and productive with the capability to maintain stable production. Additionally, the excessive use of chemical fertilizers presents a serious threat to both arable land and human health [58]. While fertilization is the major method to improve China’s AL productivity, other agrotechniques remain underutilized for improvement. The overreliance on fertilization has become one of the primary sources of pollutant emissions from agricultural production [59]. Excessive utilization of chemical fertilizers is prevalent in China, contributing to environmental challenges such as nitrate leaching, greenhouse gas emissions, phosphorus accumulation, and soil deterioration, ultimately impacting food security [60,61,62,63]. Moreover, the application of the Logarithmic Mean Divisia Index (LMDI) method has demonstrated that the current level of agricultural technological progress is insufficient to offset the combined impact of population growth and FC pattern transition on LD [26], suggesting that agricultural technology still has considerable potential for progress.

In addition, at the regional or provincial level, China has a vast territory and unbalanced regional economic development. The east has higher economic development and a higher population density compared to the west [64]. Consequently, the land demand for food consumption in the eastern region is much higher than that in the western region. Furthermore, a pressing issue in the eastern region is the acute conflict between arable land use and urbanization, with a substantial portion of arable land being consumed in the urbanization process [65]. This urbanization-induced land loss contributes to a reduction in the food supply. This imbalance between supply and demand may have a negative impact on regional food security, leading to greater food security risks in the eastern region than in the western region. On the other side of the coin, some western regions, especially Tibet and Qinghai, confront challenges due to their poor resource and environmental endowments [66], resulting in a relatively low food supply, while the land demand for food consumption in the western region will gradually increase with continuous economic development. This situation may bring challenges to food security in the western region. The interplay of these factors underscores the need for a nuanced and region-specific approach to addressing food security concerns in different parts of China.

### 4.2. Principles and Pathways for Keeping China’s Food Security from LD

Addressing the aforementioned challenges, this article proposes several key measures to ensure China's food security amidst LD under the principles of eco-efficiency, eco-effectiveness, and sufficiency: (1) healthy diet structures, (2) food waste reduction, (3) environment-friendly and sustainable agriculture improvement. These proposed measures align with the FAO recommendations that, in order to improve food security and nutrition, the world should focus on reducing the vulnerability of food production to climate change, improving food production efficiency and sustainable agricultural development capacity, and building relevant infrastructure [10,67,68,69]. Adopting comprehensive and integrated approaches based on these principles is crucial for addressing the complex interplay of factors influencing China's food security.

Promoting healthy diet structures not only has the potential to decrease LD but also contributes to keeping humans healthy [70]. The *Chinese Dietary Guide* provides recommended daily food intake ranges to guide individuals toward healthier eating habits [40]. Currently, only grain consumption in China aligns with recommendations, while dairy consumption is lower and meat consumption is higher than the recommended ranges. This high-fat diet structure may lead to high blood lipids [71,72]. If Chinese residents consume at the lower limits of the recommendation range, China’s LD for FC is estimated to be 54.6 million ha, 80% of the present value. If Chinese residents consume at the mean of the recommendation range, China’s LD for FC will be close to the present value and the health of residents will be better guaranteed, which is the optimal scenario. This optimization complies with the dual objectives of enhancing food security and promoting healthier dietary habits among Chinese residents.

Food waste leads to an increased risk of environmental impacts and resource loss and waste throughout the entire process of food production, transportation, storage, and consumption [73]. To combat this challenge, a multifaceted approach is needed. On the agricultural front, selecting high-quality germplasm and adopting advanced technologies for agricultural production, transportation, and storage can help minimize waste and loss. On the consumer side, it is necessary to encourage residents to reasonably formulate food portions and guide consumers to alter their diet structures and make appropriate food-purchasing plans. Moreover, exploring innovative business and social models, such as connecting surplus food with consumers through markets or other initiatives, can contribute to the reduction in food waste [56]. By addressing food waste comprehensively, society can not only enhance food security but also contribute to environmental sustainability and improved public health.

The profound impact of climate change on agriculture underscores the urgent need for environmentally friendly and sustainable agriculture improvements, forming the foundation of China’s food security [74]. One way to tackle this issue is by strengthening the construction of agricultural infrastructure, promoting agricultural technological progress, and guaranteeing arable land area, like by adhering to the farmland red line of 120 million hectares [75]. On the one hand, environment-friendly and sustainable agriculture could be achieved by adapting advanced agrotechniques and reducing fertilizer application. As emphasized by Liu et al., these practices can solve the negative impact of labor shortages in grain production, reduce the ecological footprint of food, and promote the sustainable development of agriculture [76]. On the other hand, scientific agricultural management such as fertilization management (e.g., soil testing formula fertilization, fertigation, and replacing chemical fertilizer with organic fertilizer), land planning and consolidation, and large-scale farming can improve the fertilizer utilization rate, soil fertility, and crop quality and reduce environmental emissions with guaranteed crop outputs [77,78,79]. In addition, integrating and coordinating relevant food and land-use practices and linking long-term goals with short-term measures and policies is much-needed, which is consistent with the policy linkage mechanism proposed by Gaupp et al. [80]. By incorporating these strategies, China can navigate the challenges posed by climate change in agriculture, paving the way for a sustainable and food-secure future.

### 4.3. Visions for Achieving China’s Food Security

Establishing laws or regulations is a decent choice for achieving food security. Currently, China has a series of programs, projects, and regulations, such as the Anti-Food Waste Law of China, the Opinions on Innovating Systems and the Mechanisms for Green Agricultural Development, and the Action for Organic Fertilizer Replaces Chemical Fertilizer, to reduce food waste and improve green agriculture. Recognizing the intrinsic link between food security, human existence, and social development, laws or regulations should be implemented as a prudent choice and should be piloted on a small scale to reduce irreversible risks caused by unsuccessful experiments. For instance, in 2015, China selected 100 counties as pilot regions to achieve fertilizer reduction through a comprehensive set of programs, policies, and regulations. Following successful outcomes in the pilot areas, those laws and regulations are intended to be introduced to other regions, demonstrating a strategic and phased approach to ensuring the efficacy and sustainability of food security measures.

Exploiting potential land resources rationally and transforming the single supply mode of cultivated land resources into a multi-supply paradigm that incorporates various types of land resources—such as woodland, grassland, desert, and lake—can alleviate the existing AL pressure [81]. Desert agriculture in Israel's Negev desert provides a good example. The desert holds abundant solar energy. If water-saving and knowledge-intensive agriculture can be developed in deserts through greenhouse application, water circulation, drip irrigation systems, and soilless culture [82,83,84], both food and land supply can be increased, demonstrating improved living standards for residents [85] and the attainment of a circular economy and economic development. This vision has been realized in the Israeli Negev desert. China possesses the world’s second-largest desert area, with deserts widely distributed in the northwest, where the existing agricultural conditions are inadequate. If desert agriculture, akin to the successful model in the Negev, can be implemented in China's desert regions, it has the potential to address the challenges of economic development in China’s desert area, increase new welfare for residents, and decrease the land pressure in China.

Indeed, food security transcends the narrow confines of food supply and consumption; it is a complex issue intertwined with multiple dimensions, such as the population, society, and environment. While China has undertaken initiatives and achieved progress in various aspects, global challenges persist, such as food waste and loss and nutrient imbalances. Consequently, addressing food security needs a more comprehensive and systematic framework that considers the intricate interplay of these multifaceted elements. Holistic approaches are crucial to navigating the challenges and ensuring sustainable and resilient food systems on a global scale.

## 5. Conclusions

The tension between social development and LD for FC has emerged as a critical factor impacting China’s food security. Although grain remains a staple in the Chinese diet, its relative share is diminishing, while the consumption and proportion of livestock products are surging. FC and LD are highly diverse among China’s districts. Residents in developed regions (e.g., the south and the east) show a preference for higher livestock product, vegetable, and fruit consumption, with a decreasing reliance on grains. At the national level, China’s LPI is low, and food security can be achieved. However, disparities in the LPI at the provincial level pose challenges, with some regions falling short of ensuring food security. In the future, China may face challenges such as increasing food consumption, diminishing AL, and severe agricultural pollution, which pose substantial risks to food security. Therefore, principles and pathways require a dual focus on both the demand and supply sides of sustainability, including but not limited to reducing food waste, promoting healthy diets, and realizing environment-friendly and sustainable agriculture. Moreover, optimizing existing laws and regulations and exploring potential land resources represent viable strategies to achieve the vision of food security.

The scope of this article, focusing on FC and AL, represents only a fraction of the multifaceted outlook of food security. Food security is a complex and interconnected system involving various dimensions of the agricultural system, such as climate, land, water, nutrients, and management, many of which are also related to planetary boundaries. In a broader context, global food security encompasses a myriad of challenges that necessitate examination within a more comprehensive and systematic framework. The considerations in this article, particularly the LPI, offer valuable insights into regional land pressure but may not fully capture the entirety of food security dynamics. Future studies should aspire to incorporate factor coupling, such as the nexus of soil, water, and climate, to provide a more holistic understanding of the intricate interplay of factors affecting food security. It is imperative to note that the lack of consideration of the global food trade in this article introduces limitations to the results. Bridging these scale gaps in further studies is essential for more comprehensive insights, as studies at the global or county scales may unveil unexpected discoveries that contribute to a more nuanced understanding of food security challenges and potential solutions. 

## Figures and Tables

**Table 1:** Food nutrient contents from FAO and food consumption range for a balanced diet.

Item			Food Nutrient Content			Balanced Range (kg·a^–1^)
			Calorie (kcal·10^2^·kg^–1^)	Protein (g·kg^–1^)	Fat (g·kg^–1^)	
Crop foods	Grain		4.65	18.86	22.34	91–146
	Vegetable		2.69	13.21	2.36	110–182
	Fruit		3.7	4.87	2.86	73–128
Livestock products	Meat	Pork	35.01	110.18	335.27	15–27
		Beef	19.74	137.97	154.03	
		Mutton	20.29	137.13	160.61	
		Poultry	17.32	118.29	135.69	
	Egg		14.72	114.59	104.6	15–18
	Dairy products		7.62	40.53	42.39	15–27
	Aquatic products		6.39	95.41	16.51	110–182

Source: Nutrient content data from the average values of various food data published in the FAO database (FAOSTAT) from 2015 to 2018 and balanced diet data from the *Chinese Dietary Guide* [40,41].

**Table 2:** Feed-to-meat ratio data of provincial districts in China.

Region	Provincial District	Pork	Beef	Mutton	Poultry	Egg	Dairy Products	Aquatic Products
Northeast	HL	3.1	2.7	4.1	2.2	2.1	0.3	1.5
	JL	2.9	2.1	2.2	2.1	1.9	0.4	1.5
	LN	2.8	2.1	2.2	2.1	1.9	0.4	1.5
North	SX	3	2.1	2.2	1.9	2	0.4	1.5
	HE	2.4	1.5	1.6	2.3	1.9	0.4	1.5
	IM	2.6	2.1	2.2	1.7	1.9	0.4	1.5
	TJ	2.7	2.1	2.2	1.9	1.8	0.4	1.5
	BJ	2.4	2.1	2.2	1.9	2	0.3	1.5
Northwest	GS	2.9	2.1	2.2	2.3	2.3	0.4	1.5
	XJ	2.4	1.7	1.9	2.3	2.3	0.3	1.5
	QJ	2.6	2.1	2.2	2.3	2.3	0.4	1.5
	NJ	2.8	2.7	2.8	2.1	2.1	0.4	1.5
	SN	3.1	2.0	2.1	2.3	2.3	0.5	1.5
Central	HA	2.9	2.0	1.5	2.1	1.9	0.4	1.5
	HN	2.7	2.1	2.2	2.3	1.9	0.4	1.5
	HB	2.4	2.1	2.2	2.5	2	0.4	1.5
East	AH	2.9	2.1	2.2	2.3	1.8	0.3	1.5
	JX	2.8	2.1	2.2	2.3	1.9	0.4	1.5
	SD	2.7	2.1	1.6	2.0	1.9	0.4	1.5
	FJ	2.9	2.1	2.2	4.1	2.0	0.4	1.5
	ZJ	2.6	2.1	2.2	2.2	1.8	0.3	1.5
	JS	2.7	2.1	2.2	2.3	1.9	0.4	1.5
	SH	2.7	2.1	2.2	2.3	1.9	0.3	1.5
South	GX	2.3	2.1	2.2	3.2	2.0	0.4	1.5
	HI	2.8	2.1	2.2	2.7	1.9	0.4	1.5
	GD	2.5	2.1	2.2	4.2	2.0	0.4	1.5
Southwest	YN	2.9	2.1	2.2	2.4	2.0	0.4	1.5
	GZ	3.1	2.1	2.2	2.3	1.9	0.6	1.5
	XZ	4.5	2.1	2.2	2.3	2.3	0.4	1.5
	SC	4.5	2.1	2.2	2.3	1.8	0.4	1.5
	CQ	2.9	2.1	2.2	2.3	2.1	0.6	1.5

Source: Data from Xin (2018) and Tang and Li (2012) [42,43].

**Table 3:** Pearson correlation analysis of per capita GDP, urbanization rate, and food consumption.

	Per Capita GDP	Urbanization Rate	Grains	Livestock Products
Per capita GDP	1 (0.000 ***)	0.877 (0.000 ***)	−0.368 (0.041 **)	0.619 (0.000 ***)
Urbanization Rate	0.877 (0.000 ***)	1 (0.000 ***)	−0.593 (0.000 ***)	0.611 (0.000 ***)
Grains	−0.368 (0.041 **)	−0.593 (0.000 ***)	1 (0.000 ***)	−0.247 (0.180)
Livestock products	0.619 (0.000 ***)	0.611 (0.000 ***)	−0.247 (0.180)	1 (0.000 ***)

Note: ***, ** represent significance levels of 1%, 5% respectively.

## Data Availability

Data are contained within the article.

## Appendix A

**Table A1:** Abbreviations and corresponding full names of provincial districts in China.

Abbreviation	Full Name	Abbreviation	Full Name
AH	Anhui	LN	Liaoning
BJ	Beijing	NJ	Ningxia
FJ	Fujian	QJ	Qinghai
GS	Gansu	SD	Shandong
GD	Guangdong	SX	Shanxi
GX	Guangxi	SN	Shaanxi
GZ	Guizhou	SH	Shanghai
HI	Hainan	SC	Sichuan
HE	Hebei	TJ	Tianjin
HA	Henan	XZ	Tibet
HL	Heilongjiang	XJ	Xinjiang
HB	Hubei	YN	Yunnan
HN	Hunan	ZJ	Zhejiang
IM	Inner Mongolia	CQ	Chongqing
JL	Jilin	MO	Macao
JS	Jiangsu	HK	Hongkong
JX	Jiangxi	TW	Taiwan

## References

[1] United Nations (2015). Transforming Our World: The 2030 Agenda for Sustainable Development. https://www.unep.org/resources/report/transforming-our-world-2030-agenda-sustainable-development.

[2] United Nations (2015). Topics-Food Security and Nutrition and Sustainable Agriculture. https://sdgs.un.org/topics/food-security-and-nutrition-and-sustainable-agriculture.

[3] Grosso G., Mateo A., Rangelov N., Buzeti T., Birt C. (2020). Nutrition in the context of the Sustainable Development Goals. Eur. J. Public Health.

[4] Li Z. (2007). Change of Chinese Inhabitant’ s Food Consumption and Nutrition Development in the Last 50 Years. Resour. Sci..

[5] Streeter L.J. (2017). Socioeconomic Factors Affecting Food Consumption and Nutrition in China: Empirical Evidence During the 1989–2009 Period. Chin. Econ..

[6] FAO, IFAD, UNICEF, WFP, WHO (2019). The State of Food Security and Nutrition in the World 2019: Safeguarding against Economic Slowdowns and Downturns.

[7] Van Dijk M., Morley T., Rau M.L., Saghai Y. (2021). A meta-analysis of projected global food demand and population at risk of hunger for the period 2010–2050. Nat. Food.

[8] OECD (2019). Agricultural Data–Meat Consumption. https://data.oecd.org/agroutput/meat-consumption.htm.

[9] OECD (2019). Health Risks–Overweight or Obese Population. https://data.oecd.org/healthrisk/overweight-or-obese-population.htm.

[10] FAO, IFAD, UNICEF, WFP, WHO (2021). The State of Food Security and Nutrition in the World 2021.

[11] FAO (2017). The Future of Food and Agriculture: Trends and Challenges. http://www.fao.org/publications/fofa/en.

[12] FAO (2018). Sustainable Development Goals. https://www.un.org/sustainabledevelopment/zh/hunger.

[13] Song G., Li M., Semakula H.M., Zhang S. (2015). Food consumption and waste and the embedded carbon, water and ecological footprints of households in China. Sci. Total Environ..

[14] Mekonnen M.M., Hoekstra A.Y. (2012). A Global Assessment of the Water Footprint of Farm Animal Products. Ecosystems.

[15] Vitali A., Grossi G., Martino G., Bernabucci U., Nardone A., Lacetera N. (2018). Carbon footprint of organic beef meat from farm to fork: A case study of short supply chain. J. Sci. Food Agric..

[16] Zhang G., Wang X., Zhang L., Xiong K., Zheng C., Lu F., Zhao H., Zheng H., Ouyang Z. (2018). Carbon and water footprints of major cereal crops production in China. J. Clean. Prod..

[17] Tilman D., Clark M. (2014). Global diets link environmental sustainability and human health. Nature.

[18] Alexander P., Brown C., Arneth A., Finnigan J., Rounsevell M.D. (2016). A. Human appropriation of land for food: The role of diet. Glob. Environ. Change.

[19] Kumwimba M.N., Meng F., Iseyemi O., Moore M.T., Bo Z., Tao W., Liang T.J., Ilunga L. (2018). Removal of non-point source pollutants from domestic sewage and agricultural runoff by vegetated drainage ditches (VDDs): Design, mechanism, management strategies, and future directions. Sci. Total Environ..

[20] Yan D., Wu S., Tang Y., Zhu J., Zhou S., Xu Z. (2022). Arable land and water footprints for food consumption in China: From the perspective of urban and rural dietary change. Sci. Total Environ..

[21] WDI Agriculture and Rural Development Data–Cultivated Land (% of Land Area). https://data.worldbank.org.cn/indicator/AG.LND.ARBL.ZS?end=2018&name_desc=false&start=2000&view=chart.

[22] Newbold T., Hudson L.N., Arnell A.P., Contu S., Palma A.D., Ferrier S., Hill S.L.L., Hoskins A.J., Lysenko I., Phillips H.R.P. (2016). Has land use pushed terrestrial biodiversity beyond the planetary boundary? A global assessment. Science.

[23] Gerbens–Leenes P.W., Nonhebel S., Ivens W.P.M.F. (2002). A method to determine land requirements relating to food consumption patterns. Agric. Ecosyst. Environ..

[24] Boyer D. (2020). Strategies for food system sustainability in China. Nat. Food.

[25] Tian T., Tang Z., Sun Y.Y. (2017). Land requirements for food in different regions of China. Acta Prataculturae Sin..

[26] Liu C., Wang F. (2018). Dynamic changes in arable land requirements for food consumption in China. Chin. J. Eco–Agric..

[27] Wang L., Anna H., Zhang L., Xiao Y., Wang Y., Xiao Y., Liu J., Ouyang Z. (2019). Spatial and Temporal Changes of Arable Land Driven by Urbanization and Ecological Restoration in China. Chin. Geogr. Sci..

[28] Liu Y., Zhou Y. (2021). Reflections on China’s food security and land use policy under rapid urbanization. Land Use Policy.

[29] He P., Baiocchi G., Hubacek K., Feng K., Yu Y. (2018). The environmental impacts of rapidly changing diets and their nutritional quality in China. Nat. Sustain..

[30] Liu R. (2014). Regional Carrying Capacity Research Based on the Improved Ecological Footprint Model. PhD. Thesis.

[31] Li Y., Wang L., Cheng S. (2019). Spatiotemporal variability in urban HORECA food consumption and its ecological footprint in China. Sci. Total Environ..

[32] Ma G. (2015). Food, eating behavior, and culture in Chinese society. J. Ethn. Foods.

[33] Monterrosa E.C., Frongillo E.A., Drewnowski A., de Pee S., Vandevijvere S. (2020). Sociocultural Influences on Food Choices and Implications for Sustainable Healthy Diets. Food Nutr. Bull..

[34] Wang Y., Wen X., Liang W., Lin X. (2023). Capital endowment, health information literacy and healthy dietary behaviors: Evidence from a survey of Chinese rural residents. Food Qual. Prefer..

[35] Zhang Y., Tian Q., Hu H., Yu M. (2019). Water Footprint of Food Consumption by Chinese Residents. Int. J. Environ. Res. Public Health.

[36] Liu X., Shi L., Engel B.A., Sun S., Zhao X., Wu P., Wang Y. (2020). New challenges of food security in Northwest China: Water footprint and virtual water perspective. J. Clean. Prod..

[37] Wackernagel M., Rees W. (1996). Our Ecological Footprint: Reducing Human Impact on the Earth.

[38] Yang L., Yu M. (2013). The Analysis of Demand on Cultivated Land of China in the Future. Econ. Geogr..

[39] d’Amour C.B., Reitsma F., Baiocchi G., Barthel S., Güneralp B., Erb K., Haberl H., Creutzig F., Seto K.C. (2017). Future urban land expansion and implications for global croplands. Proc. Natl. Acad. Sci. USA.

[40] Chinese Nutrition Society (2022). Chinese Dietary Guide, Dietary Guidelines for Chinese Residents.

[41] FAOSTAT Food and Agriculture Data. https://www.fao.org/faostat/en/#data.

[42] Xin L. (2018). Regional Production and Consumption Equilibrium of Feed grain in China and Its Policy Implication. J. Nat. Resour..

[43] Tang H., Li Z. (2012). Study on Per Capita grain Demand Based on Chinese Reasonable Dietary Pattern. Sci. Agric. Sin..

[44] (2020). China Rural Statistical Yearbook 2019: Crop Yield, Sown Area.

[45] National Bureau of Statistics of China (2020). China Statistical Yearbook 2019: Food Consumption, Permanent Population.

[46] Rae A.N. (1998). The effects of expenditure growth and urbanisation on food consumption in East Asia: A note on animal products. Agric. Econ..

[47] Godfray H.C.J., Aveyard P., Garnett T., Hall J.W., Key T.J., Lorimer J., Pierrehumbert R.T., Scarborough P., Springmann M., Jebb S.A. (2018). Meat consumption, health, and the environment. Science.

[48] Putra A.S., Tong G., Pribadi D.O. (2020). Food Security Challenges in Rapidly Urbanizing Developing Countries: Insight from Indonesia. Sustainability.

[49] Guo X., Zhang R., Xie N., Jin J. (2021). Predicting the Population Growth and Structure of China Based on Grey Fractional-Order Models. J. Math..

[50] Zhang X., Li Y., Wang Y. (2022). 14 million people Country: Towards the future of high-quality development–medium and long-term prediction of China’s population. Popul. Health.

[51] PopulationPyramid (2022). Population Pyramids of the World from 1950 to 2100. https://www.populationpyramid.net/china/2050..

[52] Sheng Y., Song L. (2019). Agricultural production and food consumption in China: A long-term projection. China Econ. Rev..

[53] OECD, FAO (2018). OECD-FAO Agricultural Outlook 2018–2027.

[54] The Wire (2022). The Enormous Scale of Global Food Waste. https://thewire.in/food/global-food-waste-index-china-india/.

[55] United Nations Environment Programme (UNEP) (2021). Food Waste Index Report 2021.

[56] Xue L., Liu X., Lu S., Cheng G., Hu Y., Liu J., Dou Z., Cheng S., Liu G. (2021). China’s food loss and waste embodies increasing environmental impacts. Nat. Food.

[57] Ministry of Agriculture and Rural Affairs of the P.R.C. (2020). 2019 National Cultivated Land Quality Grade Bulletin. https://www.moa.gov.cn/nybgb/2020/202004/202005/t20200506_6343095.htm.

[58] Van Wesenbeeck C.F.A., Keyzer M.A., Van Veen W.C.M., Qiu H. (2021). Can China’s overuse of fertilizer be reduced without threatening food security and farm incomes?. Agric. Syst..

[59] Bouwman A.F., Boumans L.J.M., Batjes N.H. (2002). Emissions of N2O and NO from fertilized fields: Summary of available measurement data. Glob. Biogeochem. Cycles.

[60] Zhang Y., Tang H., Long H., Sheng H., Huang Y., Yan X., Liao C. (2014). Eggplant and Hot Pepper’s Yield and Nutrients Uptake as Affected by Different Fertilization Structures. Hunan Agric. Sci..

[61] Wang J., Lin S., Li B. (2016). Nitrogen cycling and management strategies in Chinese agriculture. China Agric. Sci..

[62] Cui N., Cai M., Zhang X., Abdelhafez A.A., Zhou L., Sun H., Chen G., Zou G., Zhou S. (2020). Runoff loss of nitrogen and phosphorus from a rice paddy field in the east of China: Effects of long-term chemical N fertilizer and organic manure applications. Glob. Ecol. Conserv..

[63] Kalkhajeh K.Y., Huang B., Sørense H., Holm P.E., Hansen H.C.B. (2021). Phosphorus accumulation and leaching risk of greenhouse vegetable soils in Southeast China. Pedosphere.

[64] Lu Y., Zhang Y., Cao X., Wang C., Wang Y., Zhang M., Ferrier R.C., Jenkins A., Yuan J., Bailey M.J. (2019). Forty years of reform and opening up: China’s progress toward a sustainable path. Sci. Adv..

[65] Lv C., Bian B., Lee C.C., He Z. (2021). Regional gap and the trend of green finance development in China. Energy Econ..

[66] Kong D., Chen H., Wu K. (2021). The evolution of “Production-Living-Ecological” space, eco-environmental effects and its influencing factors in China. J. Nat. Resour..

[67] FAO, IFAD, UNICEF, WFP, WHO (2020). The State of Food Security and Nutrition in the World 2020; Transforming Food Systems for Affordable Healthy Diets.

[68] Zurek M., Hebinck A., Selomane O. (2022). Climate change and the urgency to transform food systems. Science.

[69] Farooq M., Rehman A., Pisante M. (2019). Sustainable Agriculture and Food Security.

[70] Dong J., Zhao Y., Wang C., Xiao X., Zhang D., Liu L., Liu X., Zhang Y., Lun F. (2019). Land demands for food consumption in Beijing during 1980–2016. Resour. Sci..

[71] Zhao B., Chen P., Zhu M. (2010). Correlations between BMI and blood pressure, blood glucose, blood lipids among male university teaching staff. China Public Health.

[72] Marotte C., Gonzales C.M.M.S., Pellegrini G.G., Friedman S.M., Lifshitz F., Mandalunis P., Zeni S.N. (2013). Low Protein Intake Magnifies Detrimental Effects of Ovariectomy and Vitamin D on Bone. Calcif. Tissue Int..

[73] Sarkar B., Debnath A., Chiu A.S.F., Ahmed W. (2022). Circular economy-driven two-stage supply chain management for nullifying waste. J. Clean. Prod..

[74] Corwin D.L. (2021). Climate change impacts on soil salinity in agricultural areas. Eur. J. Soil Sci..

[75] Liu X., He S., Chen W., Yan D., Liu L., Ding G., Zhang Z., Liu G. (2023). Strategic thinking on China’s food system transition from perspective of sustainable development goals. Bull. Chin. Acad. Sci..

[76] Liu X., Xu Y., Engel B.A., Sun S., Zhao X., Wu P., Wang Y. (2021). The impact of urbanization and aging on food security in developing countries: The view from Northwest China. J. Clean. Prod..

[77] Su C., Ma J., Chen Y. (2019). Biochar can improve the soil quality of new creation farmland on the Loess Plateau. Environ. Sci. Pollut. Res. Int..

[78] Wang C., Lv J., Xie J., Yu J., Li J., Zhang J., Tang C., Niu T., Patience B.E. (2021). Effect of slow-release fertilizer on soil fertility and growth and quality of wintering Chinese chives (*Allium tuberm* Rottler ex Spreng.) in greenhouses. Sci. Rep..

[79] Zheng E., Zhu Y., Qin M., Chen P., Liu M., Qi Z. (2023). Effects of Organic Fertilizer Replacement Nitrogen Fertilizer on Nitrogen Utilization and Growth of Mung Bean: Evidence from 15N-Tracing Technology. Agronomy.

[80] Gaupp F., Ruggeri Laderchi C., Lotze-Campen H., DeClerck F., Bodirsky B.L., Lowder S., Popp A., Kanbur R., Edenhofer O., Nugent R. (2021). Food system development pathways for healthy, nature-positive and inclusive food systems. Nat. Food.

[81] Zhu Y., Wang Z., Zhu X. (2023). New reflections on food security and land use strategies based on the evolution of Chinese dietary patterns. Land Use Policy.

[82] Mupambwa H.A., Hausiku M.K., Nciizah A.D., Dube E. (2019). The unique Namib desert-coastal region and its opportunities for climate smart agriculture: A review. Cogent Food Agric..

[83] Cao Y., Zhang W., Ren J. (2020). Efficiency Analysis of the Input for Water-Saving Agriculture in China. Water.

[84] Tal A. (2021). Israeli Agriculture—Innovation and Advancement. From Food Scarcity to Surplus.

[85] Joseph S. (2018). Farming the desert: Agriculture in the oil frontier, the case of the United Arab Emirates, 1940s to 1990s. Br. J. Middle East. Stud..

